# Circular RNA _NLRP1 targets mouse microRNA-199b-3p to regulate apoptosis and pyroptosis of hippocampal neuron under oxygen-glucose deprivation exposure

**DOI:** 10.1080/21655979.2021.1947443

**Published:** 2021-07-06

**Authors:** Bin Sun, Xiaoxian Liu, Han Peng, Xin Xiang, Hua Yang

**Affiliations:** a School of Clinical Medicine, Guizhou Medical University, Guizhou, Guiyang, P. R. China; b Department of Neurosurgery, The Affiliated Hospital of Guizhou Medical University, Guizhou, Guiyang, P. R. China; c Department of Medical Intensive Care Unit, The Affiliated Hospital of Guizhou Medical University, Guiyang, Guizhou, P. R. China

**Keywords:** circ_NLRP1, mmu-miR-199b-3p, pyroptosis, oxygen-glucose deprivation, ERK/EGR1

## Abstract

Primary hippocampal neuronal cells were used to establish cell model of cerebral ischemia under oxygen-glucose deprivation (OGD) treatment. After the cell model was pre-treated with short hairpin (sh)-circ_NLRP1 or mmu-miR-199b-3p inhibitor, LDH release and cell apoptosis were detected by LDH kit and TUNEL staining, respectively, while the expression of NLRP3 pyroptosis-related makers was analyzed through immunofluorescence (IF) assay and Western blot, respectively. The binding sites between circ_NLRP1 and mmu-miR-199b-3p were predicted and further validated by Dual Luciferase Reporter assay. Additionally, mitogen-activated protein kinase (MAPK) pathway was also analyzed by means of Western blot assay. Neuronal cells under OGD conditions released less lactate dehydrogenase (LDH) and showed less apoptosis status by silencing circ_NLRP1. In addition, gasdermin D (GSDMD)-N immunofluorescence staining showed weaker fluorescence intensity and decreased expression of pyroptosis-related mediators. We further found that mmu-miR-199b-3p-inhibitor could alter the effects of sh-circ_NLRP1 on hippocampal neuronal cells. In addition, in this process, extracellular signal-regulated kinase (ERK)/EGR1 pathway was also significantly affected. In conclusion, OGD stimulation induced neuronal damage and pyroptosis through enhancing circ_NLRP1 expression and further downregulating mmu-miR-199b-3p levels. The present study provided a novel insight for understanding the potential mechanism of ischemia-induced neuronal damage and for developing new drugs for treating brain ischemia damage.

## Graphical Abstract

**Figure:**
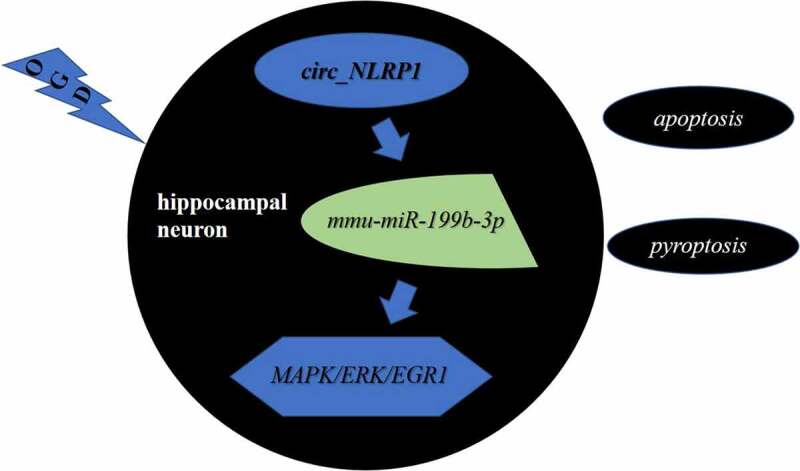

## Introduction

Ischemic brain injury occurs when a thrombosis or embolism in the brain causes a blockage of a certain vessel, resulting in reduced blood flow and inadequate oxygen supply to the affected area. In acute cerebral ischemia, the assessment of irreversible injury is critical for treatment options and patient prognosis. Recent studies have shown that rapid and reliable ncRNA screening is helpful for early diagnosis and prognosis of stroke. Compared with miRNA and lncRNA, circRNA has a relatively long half-life and thus is widely recognized as an ideal biomarker for the diagnosis and prognosis of stroke [1–3]. Pyroptosis, which plays a vital role in ischemic brain injury, occurs in neurons and endothelial cells [4–6]. MCC950, an inhibitor of NLRP3, can significantly reduce the expression of NLRP3, caspase 1 and IL 1β in the hypopenumbral zone. Furthermore, its functional role in improving the neurological function score of mice and reducing the cerebral infarction area and the degree of brain tissue edema has been also been mentioned by a previous report [7]. NLRP3 knockdown after cerebral ischemia can significantly reduce cerebral infarction and damage to the blood-cerebrospinal fluid barrier and improve the brain damage to mice after cerebral ischemia [8]. These data indicated an important role of pyroptosis in cerebral ischemia.

Different from linear RNA, circRNAs, a new type of endogenous non-coding RNA, are characterized by a covalently closed-ring structure and lack either a 5ʹ-terminal cap structure or a 3ʹ-terminal poly (adenosine) tail [9]. Accumulating evidence has indicated an altered expression of circRNAs in ischemic stroke models, suggesting that circRNAs may be involved in the pathophysiological process of stroke [10]. Some studies found that circRNA may act as a miRNA sponge or a competitive endogenous RNA (ceRNA) to bind to regulate the expression of miRNA, and then modulate the expression level of a certain target gene [11,12]. A study found that miRNA played a vital role in suppressing neuron apoptosis induced by cerebral ischemia/reperfusion injury [13]. After middle cerebral artery induction, miR-199b-3p expression showed downregulation in brain tissues, MAPK/ERK/EGR1 axis was activated and cerebral microvascular endothelial cells showed increased apoptosis. The study indicated that miR-199b-3p up-regulation could inhibit the apoptosis of cerebral microvascular endothelial cells by blocking MAPK/ERK/EGR1 axis, thus protecting mice from ischemic stroke [14]. Based on the literature reviewing, we predict that there may exist an association of circ_NLRP1 with miR-199b-3p involving in ischemia-induced neuron injury, which could be related to MAPK/ERK/EGR1 axis. The study was designed to investigate the regulatory role of circ_NLRP1 in OGD-induced mouse primary hippocampal neurons and whether mmu-miR-199b-3p partook in its regulatory role to shed light on the role of circ_NLRP1/mmu-miR-199b-3p in hippocampal neurons under OGD induction.

## Methods

### Mouse model of transient focal cerebral ischemia

In male mice, transient focal cerebral ischemia was caused by middle cerebral artery occlusion (MCAO) [15]. Mice were randomly divided into sham group and model group, and anesthetized with isoflurane (3% induction, 1.0% ~ 1.5% maintenance) mixed with oxygen and nitrogen. From the right common carotid artery, the thread (Guangzhou Jialing Biotechnology Co., Ltd., Guangzhou, China) was inserted into carotid endarterectomy and finally into the middle cerebral artery until resistance was felt (8 − 12 mm). After 1 h of ischemia, the thread was removed for reperfusion. After 24 h, the hippocampal region was removed and collected. The study was approved by the ethical committee of The Affiliated Hospital of Guizhou Medical University.

#### OGD assay

Primary neurons of the CA1 hippocampus from E12-E14 embryos of C57BL/6 J mice were isolated, and CA1 hippocampal neurons were seeded into serum-free neuron basal culture medium containing 2% B27 supplement and 2 mM glutamine. On day 8 in vitro, neurons were induced with OGD [16]. Hippocampal neuronal cells were cultured in medium containing no glucose in incubator with 94% N_2_, 5% CO_2_ and 1% O_2_.

#### Quantitative real-time PCR (qPCR)

After OGD treatment, total RNA was extracted and reversely transcribed into cDNA (a HiScript Q RT SuperMix, Vazyme, Nanjing, China) for the analysis of circ_NLRP1 expression. Next, the circ_NLRP1 expression was detected using SYBR Green Real-time PCR Master Mix (TOYOBO, Japan). Next, for mmu-miR-199b-3p expression analysis, total RNA was reversely transcribed into cDNA using All-in-one^TM^ miRNA First-strand cDNA synthesis kit (GeneCopoeia) and quantified using miScript SYBR® Green PCR Kit (Vazyme, QIAGEN). GAPDH and U6 were, respectively, taken as the internal reference of circ_NLRP1 and mmu-miR-199b-3p. The relative expression of circ_NLRP1 or mmu-miR-199b-3p was calculated by 2^−ΔΔCt^. The experiment was repeated for three times. The primers used in this experiment are as follows: circ_NLRP1, Forward Primer: 5ʹ-GGACCTCATGGTGGTTACTTTC-3, Reverse Primer: 5ʹ-TCCCAGGGGCCGTAAACTT-3ʹ. mmu-miR-199b-3p, Forward Primer 5ʹ-: GTCACAGTAGTCTGCACAT-3ʹ, Reverse Primer: GTGCAGGGTCCGAGGT-3ʹ. U6, Forward Primer: 5ʹ-TCCGACGCCGCCATCTCTA-3ʹ, Reverse Primer: 5ʹ -TATCGCACATTAAGCCTCTA-3ʹ. Caspase 1, Forward Primer: 5ʹ-ACAAGGCACGGGACCTATG-3ʹ, Reverse Primer: 5ʹ-TCCCAGTCAGTCCTGGAAATG-3ʹ. GSDMD, Forward Primer: 5ʹ-ATGCCATCGGCCTTTGAGAAA-3ʹ, Reverse Primer: 5ʹ-AGGCTGTCCACCGGAATGA-3ʹ. Pannexin-1, Forward Primer: 5ʹ-CCACCGAGCCCAAGTTCAA-3ʹ, Reverse Primer: 5ʹ-GGAGAAGCAGCTTATCTGGGT-3ʹ. NLRP3, Forward Primer: 5ʹ-ATTACCCGCCCGAGAAAGG-3ʹ, Reverse Primer: 5ʹ-CATGAGTGTGGCTAGATCCAAG-3ʹ. GAPDH, Forward Primer: 5ʹ-AGGTCGGTGTGAACGGATTTG-3ʹ, Reverse Primer: 5ʹ-GGGGTCGTTGATGGCAACA-3ʹ.

#### MTT assay

After cells were exposed to OGD for 2 h, 4 h or 6 h, MTT solution of 20 μL was added and incubated with cells for 4 h. The absorbance at 490 nm was detected. The experiment was repeated for three times with three multiple holes each time.

#### Plasmids transfection

Lipofectamine 3000 was used to perform transfection experiment. The primary hippocampus neurons were transfected with shRNA-circ_NLRP1 or shRNA-NC lentivirus, or mmu-miR-199b-3p mimic or mmu-miR-199b-3p-inhibitor for 24 h before OGD treatment (HANBIO, Shanghai, China).

#### LDH kit

After the cell treatment, the supernatant was collected in the new 96-well plate, and the activity of LDH determined by microplate method (Nanjing Jiancheng Bioengineering institute, Nanjing, China). Six multiple wells were set for each concentration, and the experiment was repeated for three times.

#### TUNEL staining

After the primary neurons were transfected and then treated with OGD/R, the cells were fixed with 4% paraformaldehyde at room temperature for 20 min, washed with PBS for three times each time for 5 min. According to the instructions of the TUNEL cell apoptosis detection kit (Jiangsu KeyGEN BioTECH Corp., Ltd., Nanjing, China), corresponding reagents were successively added and cells were photographed under a fluorescence microscope (Olympus). The experiment was repeated for three times.

#### Immunofluorescence (IF) staining

After being dried, cell slides were fixed with ice methanol for 15 min, incubated with 0.25% Triton X-100/PBS at room temperature for 15 min and sealed with 10% BSA-/PBS for 1 h. Primary antibody (Abcam, England) was added for incubation overnight at 4°C. Then, secondary antibody was added to incubate for 1 h at room temperature. After being rinsed with PBS, the sections were incubated with DAPI (1 g/ml) at room temperature and in the dark for 10 min. After being rinsed with PBS, the sections were sealed with glycerinum and photographed using a fluorescence microscope (Olympus). The experiment was repeated for three times.

#### Western blot

The protein levels of caspase 1, GSDMD-N, Pannexin-1, NLRP3 and pro-caspase 1 were measured by the following steps. Total proteins were extracted, quantified by BCA method, transferred to PVDF by electrophoresis, blocked for 2 h and incubated overnight with primary antibodies. Quantity One software was used to measure the gray value of each strip. The ratio of the target strip to the GAPDH strip was taken as the relative expression level of proteins. The experiment was repeated for three times.

#### Luciferase reporter gene assay

Wild-type (WT) and mutant-type (Mut) circ_NLRP1 sequences were cloned into pGL3 plasmid, respectively. HEK293T cells were co-transfected with the reporter vector and mmu-miR-199b-3p mimics, and luciferase activity was finally determined with the dual-luciferase reporter assay kit (Promega). The experiment was repeated for four times.

#### Statistical analysis

The statistical analysis was performed using GraphPad 7.0 software. The measurement data were expressed as mean±standard deviation, and the comparison between groups was performed by ANOVA and *t*-test. P < 0.05 was considered as statistically significant. All experiments were at least repeated three times.

## Results

### The effects of OGD treatment in hippocampal neurons

**Figure 1.**
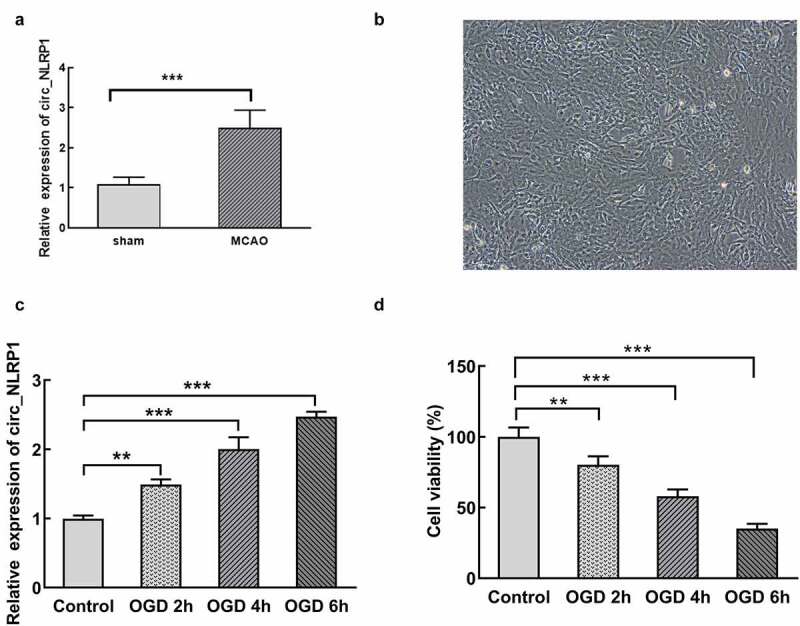
MCAO and OGD treatment increased circ_NLRP1 levels and OGD treatment reduced cell viability in primary hippocampal neurons in time-dependent manner. The expression of circ_NLRP1 through the analysis of RT-qPCR, n = 6. (a). Light microscopy of hippocampal neurons(A). The expression analysis of circ_NLRP1 through RT-qPCR (b). The detection of cells viability through CCK8 assay (c). **p < 0.01, ***p < 0.001

To explore the role of circ_NLRP in vitro model of ischemic stroke, the OGD model of mouse hippocampal neurons first was established. Of note, the level of circ_NLRP1 MCAO treatment markedly induced an increase in hippocampus of mice when compared with sham group (Figure 1a). Hippocampal neuron is shown in Figure 1b. After mouse hippocampal neurons were exposed to OGD treatment for 2, 4 or 6 h, respectively, increased circ_NLRP1 expression levels by qPCR analysis were found in a time-dependent manner (Figure 1c). The results implied a potential relationship between OGD induction and circ_NLRP1. OGD stimulation for different durations significantly inhibited cell viability of hippocampal neurons (Figure 1d). Specifically, OGD treatment for 4 h nearly led to half of the cell death, and another 2 h treatment induced more aggravated cell damage.

Circ_NLRP1 silencing significantly decreased neuronal damage under OGD conditions.

**Figure 2.**
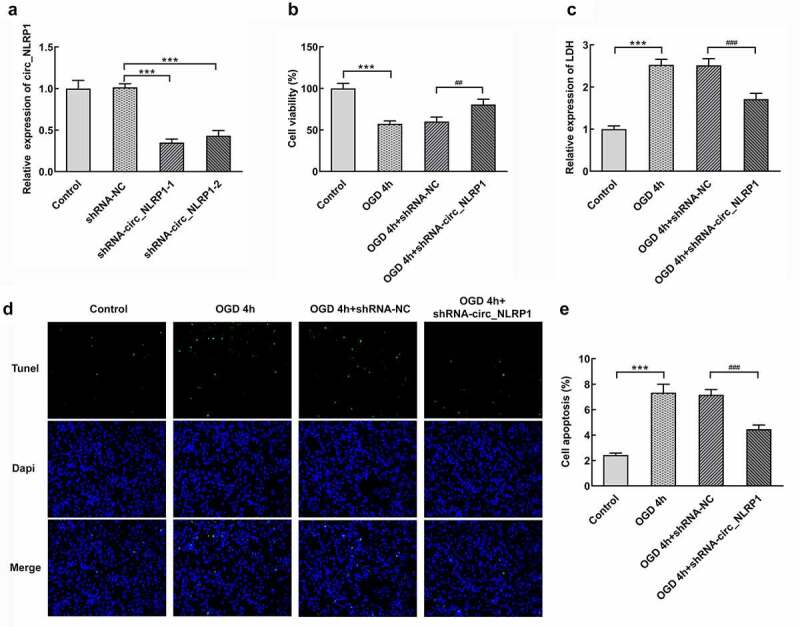
The effects of circ_NLRP1 on viability, LDH release and apoptosis in mouse hippocampal neuronal cells treated by OGD. The transfection efficacy of shRNA-circ_NLRP1-1 or shRNA-circ_NLRP1-2 by qPCR analysis (a). The analysis of cell viability through MTT assay (b), the LDH release through LDH kit (c), cell apoptosis through TUNEL kit (d–e) in cells undergoing shRNA-circ_NLRP1-1 or shRNA-circ_NLRP1-2 transfection for 24 h and then OGD treatment for 4 h. ***p < 0.001. ^##^p < 0.01, ^###^p < 0.001

In order to further determine whether circ_NLRP1 is involved in neuronal damage induced by OGD, we transfected mouse hippocampal neuronal cells with circ_NLRP1 knockdown plasmids (shRNA-circ_NLRP1-1 or shRNA-circ_NLRP1-2) and detected the transfection efficiency. As shown in Figure 2a, the expression of circ_NLRP1 was significantly reduced after transfection. Hippocampal neuronal cells with shRNA-circ_NLRP1-1 transfection expressed lower circ_NLRP1 levels than cells with shRNA-circ_NLRP1-2 transfection (Figure 2a). Next, we utilized shRNA-circ_NLRP1-1 to knock down shRNA-circ_NLRP1 and mouse hippocampal neuronal cells were transfected with shRNA-circ_NLRP1 for 24 h and then exposed to OGD for 4 h. MMT solution was mixed with cells to evaluate cell activities. circ_NLRP1 knockdown significantly suppressed the influence of OGD stimulation on cell viability (Figure 2b). Cell supernatant was collected to detect LDH activities for the analysis of the degree of cell damage. We found that circ_NLRP1 knockdown could markedly reduce LDH release induced by OGD for 4 h, suggesting that circ_NLRP1 silencing protected neurons against OGD-induced damage (Figure 2c). Next, we further observed that hippocampal neuronal cells exhibited obvious apoptosis after OGD exposure than control group (Figure 2d-e).

Circ_NLRP1 silencing significantly reduced pyroptosis mediators in hippocampal neuronal cells OGD conditions.

**Figure 3.**
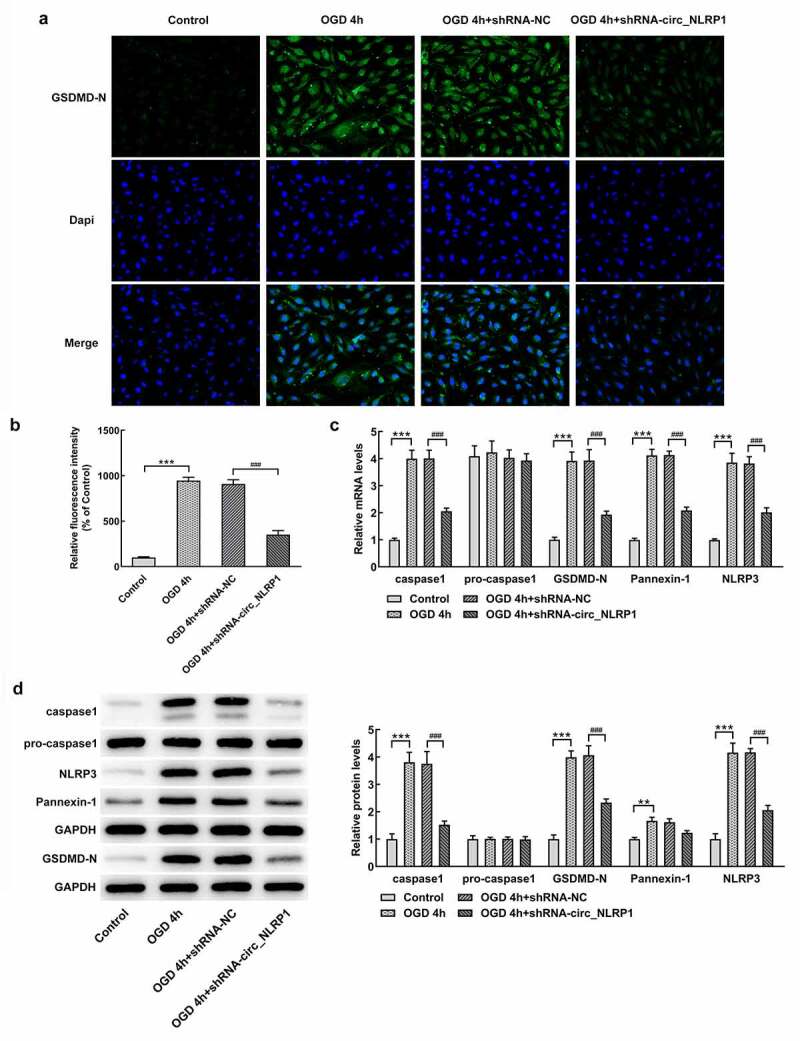
The effects of circ_NLRP1 on NLRP3-mediated pyroptosis pathway in OGD-induced in mouse hippocampal neuronal cells. The immunofluorescence analysis of GSDMD-N (a–b). The RT-qPCR (b) and the western blotting analysis (c–d) of pyroptosis-related proteins and pannexin-1. ***p < 0.001, ^###^p < 0.001

Next, we wonder to know whether pyroptosis is implicated in the regulatory role of circ_NLRP1 in response to OGD. The GSDMD-N terminal domain can target the cell membrane, bind to the phospholipid protein on the cell membrane, polymerize and form pores in the cytoplasmic membrane, thus inducing the pyroptosis of cells. OGD stimulation for 4 h markedly induced the production of GSDMD-N in mouse hippocampal neuronal cells (Figure 3a-b). The protein levels of GSDMD-N were significantly decreased by silencing circ_NLRP1 as compared to OGD treatment group or 4 h. NLRP3 inflammasome complex was evidenced to be associated with neuronal damage partly mediated by the activation of caspase-1 [17,18]. The NLRP3 inflammasome-related proteins, NLRP3 and caspase 1, as well as pyroptosis executor, GSDMD-N showed increased levels and, after OGD stimulation for 4 h, no significant change in pro-caspase 1 was observed. These data could imply the activation of NLRP3 pyroptosis pathway by OGD stimulation. Surprisingly, these effects induced by OGD stimulation were significantly dampened after pre-treatment of shRNA-circ_NLRP1 plasmids. Additionally, we also observed similar influence on pannexin-1 through OGD treatment and circ_NLRP1 silencing (Figure 3 C-D).

circ_NLRP1 bound to mmu-miR-199b-3p and regulated its expression.

**Figure 4.**
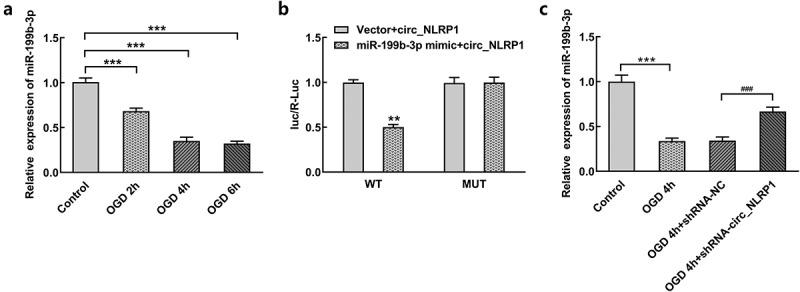
Effect of circ_NLRP1 silencing on expression of mmu-miR-199b-3p in mouse hippocampal neuronal cells under OGD stimulation. The upregulation of mmu-miR-199b-3p expression after OGD treatment. The result of luciferase assay (b). The elevation of mmu-miR-199b-3p expression after circ_NLRP1 silencing (c). ***p < 0.001, ^###^p < 0.001

CircRNA has been investigated to function as a sponge of miRNA to regulate the expression of its target gene. There may be binding sites between circ_NLRP1 and mmu-miR-199b-3p through RNAhybrid 2.2 database (https://bibiserv.cebitec.uni-bielefeld.de/rnahybrid). In order to study the regulatory role of circ_NLRP1, we further examined the expression of mmu-miR-199b-3p in OGD-induced mouse hippocampal neuronal cells. OGD treatment has obvious inhibitory effects on the expression of mmu-miR-199b-3p in a time-dependent manner (Figure 4a). Next, we inserted the full circ_NLRP1 sequence into the luciferase report and found that luciferase activity of wild type (circ_NLRP1 WT) significantly decreased after mmu-miR-199b-3p mimics (Figure 4b). However, no significant changes in the activity of the mutant (circ_NLRP1 Mut) were observed compared with the control group, suggesting their direct binding association. We transfected the circ_NLRP1 knockdown plasmid to silence circ_NLRP1 and further detected the effect on the expression of mmu-miR-199b-3p. As shown in Figure 4c, after circ_NLRP1 silencing, the expression level of mmu-miR-199b-3p was significantly increased.

mmu-miR-199b-3p-inhibitor suppressed the effects of sh-circ_NLRP1 on cell viability and apoptosis in mouse hippocampal neuronal cells stimulated by OGD.

**Figure 5.**
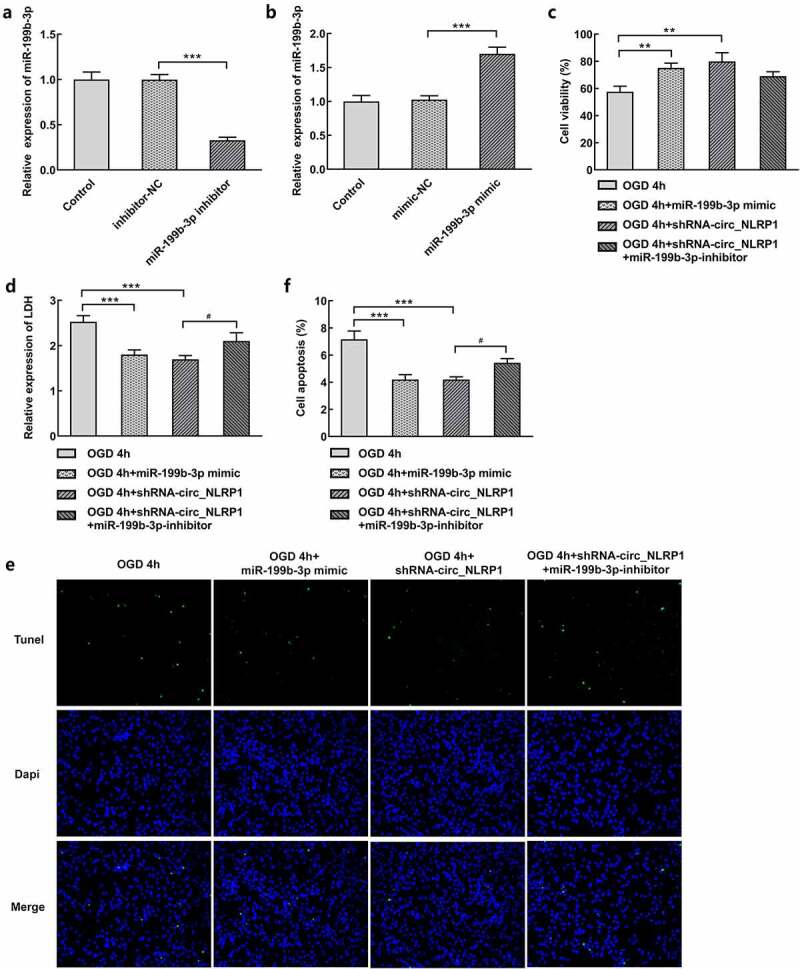
Mmu-miR-199b-3p mediated the function of circ_NLRP1. The effects of mmu-miR-199b-3p-inhibitor through qPCR analysis (a). The effects of mmu-miR-199b-3p-mimic through qPCR analysis (b). mmu-miR-199b-3p-inhibitor disturbed the effects of shRNA-circ_NLRP1 on cell viability (c), LDH (d) or cell apoptosis (e–f). **p < 0.01, ***p < 0.001. ^#^p < 0.05

In this step, we mainly focused on whether mmu-miR-199b-3p mediated the function of circ_NLRP1 in OGD-stimulated mouse hippocampal neuronal cells. mmu-miR-199b-3p-inhibitor or mmu-miR-199b-3p-mimic was, respectively, used to silence or overexpress mmu-miR-199b-3p. qPCR result showed that they exerted significant effects on suppressing mmu-miR-199b-3p expression or promoting mmu-miR-199b-3p expression (Figure 5a-b). Furthermore, mmu-miR-199b-3p mimic significantly enhanced cell activities than model group (Figure 5c). Co-transfection with shRNA-circ_NLRP1 and mmu-miR-199b-3p-inhibitor in primary hippocampal neuronal cells resulted in the elimination of the promoting effects on cell viability caused by knockdown of circ_NLRP1 alone. Additionally, mmu-miR-199b-3p mimic significantly suppressed LDH release and apoptosis levels compared with model group (Figure 5d-f). Moreover, mmu-miR-199b-3p-inhibitor could markedly disturb the effects of shRNA-circ_NLRP1 in primary hippocampal neuronal cells exposed to OGD for 4 h.

circ_NLRP1 changed the effects of sh-circ_NLRP1 on NLRP3-mediated pyroptosis.

**Figure 6.**
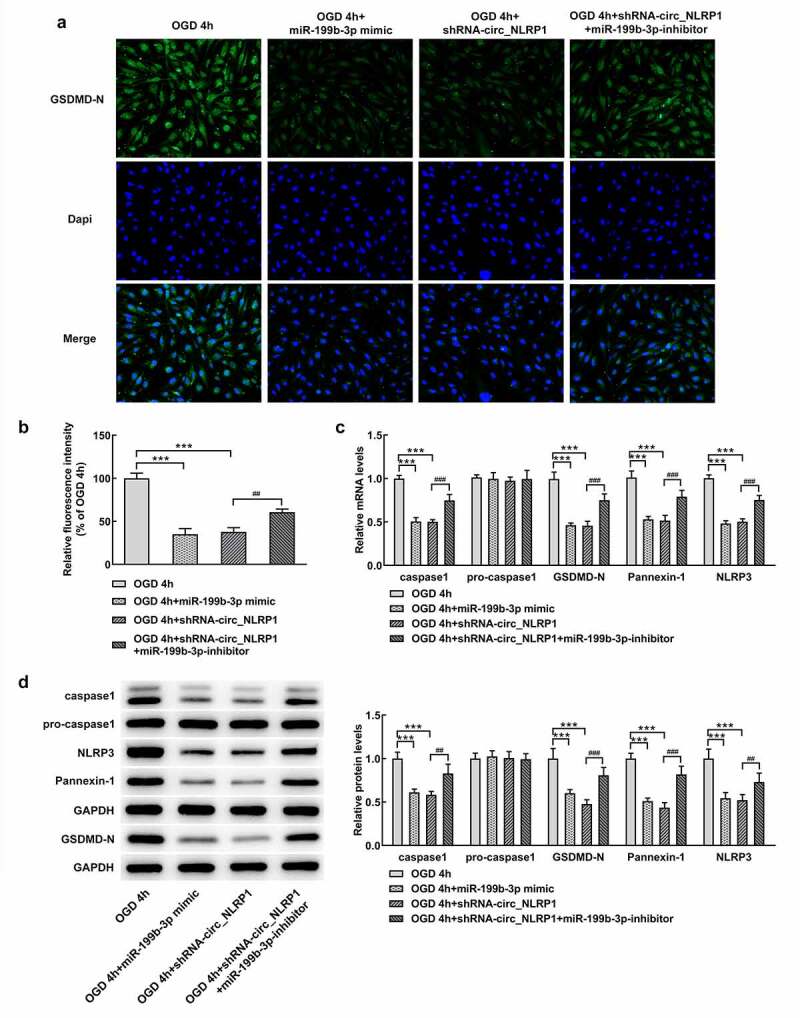
The regulation of circ_NLRP1 for pyroptosis via circ_NLRP1

**Figure 7.**
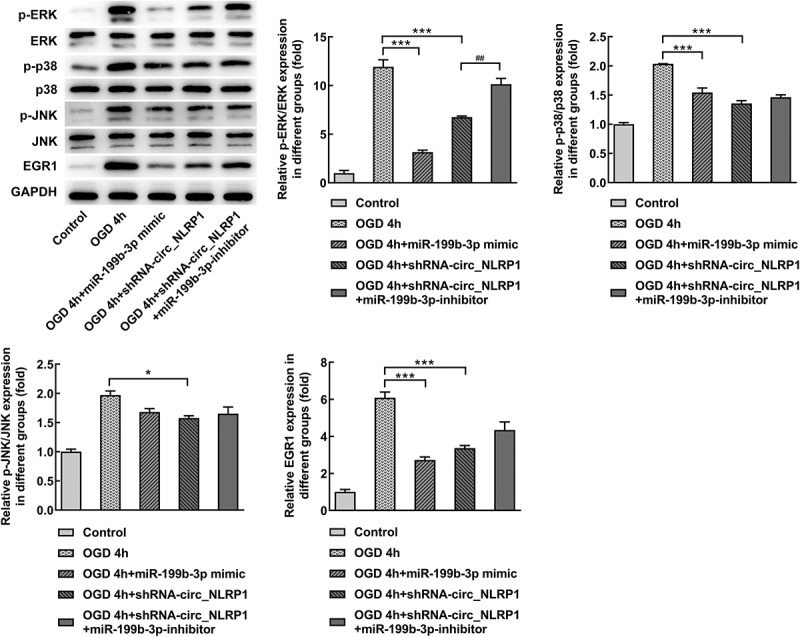
The regulation of circ_NLRP1 fo ERK/EGR1 pathway via circ_NLRP1. The analysis of MAPK pathway through Western blotting. *p < 0.01, ***p < 0.001. ^##^p < 0.05

To further analyze whether mmu-miR-199b-3p also mediated the function of circ_NLRP1 in pyroptosis. As shown in Figure 6a, GSDMD-N expression was significantly decreased after mmu-miR-199b-3p overexpression. After cells were co-transfected with mmu-miR-199b-3p mimic and shRNA-circ_NLRP1 plasmid, GSDMD-N expression was obviously increased (Figure 6a-b). We further analyzed the expression levels of pyroptosis-related proteins, which are caspase 1, GSDMD-N and NLRP3. Intriguingly, the expression of caspase 1, GSDMD-N, Pannexin-1 and NLRP3 was significantly suppressed after mmu-miR-199b-3p ovexpression (Figure 6c-d). Moreover, mmu-miR-199b-3p-inhibitor significantly decreased the inhibitory effects of shRNA-circ_NLRP1 on these, indicating that mmu-miR-199b-3p could mediate the effects of shRNA-circ_NLRP1 on pyroptosis. Next, MAPK pathway was investigated to confirm its participation in circ_NLRP1. OGD treatment significantly increased the levels of p-ERK, p-p38, p-JNK and EGR1, but mmu-miR-199b-3p mimic could markedly lessen these effects. Additionally, mmu-miR-199b-3p-inhibitor significantly relieved the effects of shRNA-circ_NLRP1 on p-ERK and EGR1 (Figure 7). However, mmu-miR-199b-3p-inhibitor did not significantly alter the effects of shRNA-circ_NLRP1 on the phosphorylated levels of p38 and JNK. These data could imply that the involvement of ERK/EGR1 pathway in regulatory role of circ_NLRP1/ mmu-miR-199b-3p.

Representative images showing immunoreactivity to GSDMD-N (green) in primary hippocampal neuronal cells (A–B). The analysis of pyroptosis-related protein expression through qPCR. **p < 0.01, ***p < 0.001. ^##^p < 0.05, ^###^p < 0.001.

## Discussion

Ischemic cerebrovascular disease is the disease with one of the world’s highest morbidity, disability and mortality, posing a serious threat to the health and quality of life of the middle-aged and the elderly [19]. In the present study, increased circ_NLRP1 levels were observed in mouse hippocampus with MCAO treatment and hippocampal neuronal cells under OGD conditions. Further mechanistical studies revealed that circ_NLRP1 silencing protected neuron against LDH release and cell apoptosis induced by OGD.

Rapid cell death could be seen in ischemic stroke where amounts of nutrients were lost, accompanied by a series of harmful pathophysiological events [20–22]. In our work, circ_NLRP1 was identified to play a potential regulatory role during the pathophysiological process of ischemic stroke, as demonstrated by our data that showed the initiation of cell apoptosis could be evoked by increased circ_NLRP1 under OGD treatment or NLRP3 mediation. Specifically, NLRP3-mediated pyroptosis mediators were found to be elevated in OGD-treated PC12 cells [23]. Additionally, some other cell types also exhibited NLRP3-mediated pyroptosis under oxygen-glucose deprivation, such as myocardial cells and alveolar macrophages [24,25]. Circular RNA was widely investigated in ischemic stroke and affected cerebral ischemic injury, the function of which was mediated by targeting miRNA [3,26,27]. Interestingly, the experiment further confirmed the complementary sites between circ_NLRP1 and mmu-miR-199b-3p and their negative association as circ_NLRP1 silencing contributed to a marked upregulation of mmu-miR-199b-3p expression. Besides, mmu-miR-199b-3p overexpression decreased LDH release, cell apoptosis and pyroptosis, while mmu-miR-199b-3p inhibitor markedly altered the effects of shRNA-circ_NLRP1 in these cell processes. The regulatory role of microRNA-199b-3p in cell apoptosis has been validated in some studies. A research reported that cerebral microvascular endothelial cells displayed less apoptosis levels through microRNA-199b-3p via downregulating MAPK/ERK/EGR1 axis in ischemic stroke [14]. In addition, microRNA-199b-3p upregulation induced apoptosis mediated by mitochondria pathway through targeting ZEB1 [28]. Then, MAPK pathway was further investigated in hippocampus neurons under OGD conditions where mmu-miR-199b-3p inhibitor or shRNA-circ_NLRP1 was introduced. We observed that ERK/EGR1 axis was markedly regulated via circ_NLRP1/mmu-miR-199b-3p. Therefore, following OGD treatment, circ_NLRP1/mmu-miR-199b-3p axis could further induce neuronal apoptosis by affecting the activities of ERK/EGR1 axis. To sum up, the downregulation of circ_NLRP1 protected neurons against apoptosis and pyroptosis induced by OGD via mmu-miR-199b-3p. Our study reveals the role of circ_NLRP1 mediated by mmu-miR-199b-3p in pyroptosis in hippocampal neurons with OGD treatment for the first time, providing a new insight for the pathomechanism of ischemic brain injury.

### Conclusion

Circ_NLRP1/mmu-miR-199b-3p affects pyroptosis in hippocampal neurons under OGD challenge, which could be related to ERK/EGR1 axis.

## Data Availability

The experimental data were available from correspondence author based on a reasonable request.

## Highlights

1. Circ_NLRP1 levels showed upregulation in vivo and in vitro models of ischemic stroke.

2. Oxygen-glucose deprivation stimulated hippocampal neurons expressing increased circ_NLRP1 to lead to cell pyroptosis by mmu-miR-199b-3p.

3. Targeting circ_NLRP1/mmu-miR-199b-3p could be a promising strategy for ischemic brain injury.
